# Supplementary material for: What’s in a Name? Experimental Evidence of Gender Bias in Recommendation Letters Generated by ChatGPT
Source: J Med Internet Res. 2024 Mar 5;26:e51837. doi: 10.2196/51837 (PMC10951834; doi:10.2196/51837)
Supplement: Multimedia Appendix 1 [file jmir_v26i1e51837_app1.docx]

**Supplementary Document 1 (results of studies 1 and 2)**

*Table S1. Percentages of comparisons that yielded significant results according to prompts and hypothesized directions*

|  | Study 1 Prompts | | | Study 2 Prompts | | |
| --- | --- | --- | --- | --- | --- | --- |
|  | A | B | C | A^1^ | B^1^ | C^1^ |
| Significant comparisons | 64.3% | 35.7% | 53.6% | 46.4% | 60.7% | 85.7% |
| % in favor of H | 33.3%* | 100% | 86.7% | 69.2%* | 64.7% | 41.7% |
| % against H | 66.7%* | 0% | 13.3% | 30.8%* | 35.3% | 48.3% |

*Note.* H = hypothesis, * = No differences were hypothesized. There were no *a priori* hypothesized differences for prompts A and A^1^ (unlike the other prompts tested, for which gender-based differences in language were hypothesized). However, many gender-based differences in language emerged for prompts A and A^1^ as well. Percentages are therefore reported for consistency with hypotheses H2B and H2C, which were applied for prompts B, B^1^, C, and C^1^, for easy comparison of overall trends across prompts.

*Table S2. Independent samples t tests for prompt A (“research position”)*

|  | M(SD) | |  |  |  | 95% CI | |
| --- | --- | --- | --- | --- | --- | --- | --- |
| Variable | Male | Female | F - M | *t* | *p* | UL | LL |
| Word Count | 451.13(43.77) | 442.61(28.20) | -8.52 | -1.636 | 0.104 | 1.758 | -18.798 |
| Include | 1.88(0.39) | 1.95(0.42) | 0.07 | 1.291 | 0.198 | 0.188 | -0.039 |
| Agentic | 3.28(0.71) | 3.20(0.60) | -0.08 | -0.940 | 0.349 | 0.096 | -0.271 |
| Agentic+Include | 3.89(0.62) | 4.00(0.71) | 0.11 | 1.162 | 0.247 | 0.295 | -0.076 |
| Avoid | 0.61(0.23) | 0.72(0.22) | 0.11 | 3.474 | <.001 | 0.173 | 0.048 |
| Communal | 0.61(0.35) | 0.52(0.33) | -0.09 | -1.951 | 0.052 | 0.001 | -0.189 |
| Communal+Avoid | 1.22(0.37) | 1.23(0.37) | 0.01 | 0.227 | 0.821 | 0.115 | -0.091 |
| Analytic | 73.66(5.20) | 74.18(6.65) | 0.52 | 0.616 | 0.538 | 2.186 | -1.145 |
| Clout | 81.21(5.51) | 84.19(5.45) | 2.98 | 3.846 | <.001 | 4.510 | 1.453 |
| Personal Pronouns | 10.37(0.79) | 10.60(0.93) | 0.23 | 1.922 | 0.056 | 0.475 | -0.006 |
| Negation | 0.45(0.16) | 0.25(0.26) | -0.20 | -6.423 | <.001 | -0.134 | -0.253 |
| Auxiliary Verbs | 5.38(0.85) | 4.81(1.04) | -0.57 | -4.228 | <.001 | -0.303 | -0.832 |
| Standard Verbs | 9.46(0.91) | 8.74(1.18) | -0.72 | -4.785 | <.001 | -0.419 | -1.007 |
| Adjectives‡ | 7.47(0.73) | 7.22(0.85) | -0.25 | -2.233 | 0.027 | -0.029 | -0.471 |
| Tentative | 1.84(0.46) | 1.74(0.42) | -0.10 | -1.587 | 0.114 | 0.024 | -0.233 |
| Affiliation | 2.86(0.55) | 3.00(0.69) | 0.14 | 1.505 | 0.134 | 0.305 | -0.041 |
| Achievement | 7.83(0.95) | 8.44(1.20) | 0.61 | 3.959 | <.001 | 0.904 | 0.303 |
| Emotion | 1.17(0.39) | 1.30(0.40) | 0.13 | 2.429 | 0.016 | 0.247 | 0.026 |
| Positive emotion | 1.13(0.39) | 1.27(0.39) | 0.14 | 2.414 | 0.017 | 0.243 | 0.025 |
| Social behavior | 6.95(1.05) | 6.52(0.83) | -0.43 | -3.222 | 0.002 | -0.167 | -0.694 |
| Prosocial behavior | 2.64(0.51) | 2.54(0.48) | -0.10 | -1.494 | 0.137 | 0.033 | -0.241 |
| Politeness | 0.57(0.16) | 0.76(0.30) | 0.19 | 5.393 | <.001 | 0.250 | 0.116 |
| Moralization | 0.55(0.26) | 0.41(0.29) | -0.14 | -3.541 | <.001 | -0.059 | -0.210 |
| Communication | 2.03(0.53) | 1.67(0.44) | -0.36 | -5.073 | <.001 | -0.215 | -0.489 |
| Social referents | 10.39(1.17) | 11.17(1.22) | 0.78 | 4.564 | <.001 | 1.107 | 0.439 |
| Reward | 0.49(0.29) | 0.54(0.26) | 0.05 | 1.249 | 0.213 | 0.127 | -0.028 |
| Risk | 0.21(0.09) | 0.11(0.12) | -0.10 | -7.057 | <.001 | -0.076 | -0.135 |
| Curiosity | 4.01(0.59) | 4.05(0.64) | 0.04 | 0.389 | 0.698 | 0.205 | -0.138 |
| Need | 0.62(0.20) | 0.63(0.17) | 0.01 | 0.326 | 0.745 | 0.061 | -0.043 |

*Note.* + = higher score for historically female name
‡ = no longer significant after Benjamini-Hochberg step-up correction.

*Table S3. Independent samples t tests for prompt B (“early career award”)*

|  | **M(SD)** | |  |  |  | **95% CI** | |
| --- | --- | --- | --- | --- | --- | --- | --- |
| **Variable** | **Male** | **Female** | **F - M** | ***t*** | ***p*** | **UL** | **LL** |
| Word Count | 435.91(42.23) | 450.25(37.89) | 14.34 | 2.527 | 0.012 | 25.529 | 3.151 |
| Include | 1.66 (0.51) | 1.75(0.45) | 0.09 | 0.951 | 0.343 | 0.199 | -0.070 |
| Agentic | 3.45(.62) | 3.16(.65) | -0.29 | -3.296 | 0.001 | -0.119 | -0.472 |
| Agentic+Include | 4.09(.71) | 3.85(.71) | -0.24 | -2.406 | 0.017 | -0.044 | -0.439 |
| Avoid | 0.70(0.21) | 0.65(0.20) | -0.05 | -1.474 | 0.142 | 0.014 | -0.099 |
| Communal | 0.47(0.39) | 0.40(0.30) | -0.07 | -1.374 | 0.171 | 0.029 | -0.165 |
| Communal+Avoid‡ | 1.16(0.42) | 1.04(0.33) | -0.12 | -2.163 | 0.032 | -0.010 | -0.222 |
| Analytic | 73.94(6.20) | 69.55(6.02) | -4.39 | -5.075 | <.001 | -2.681 | -6.090 |
| Clout | 77.80(7.31) | 86.39(5.81) | 8.59 | 9.196 | <.001 | 10.433 | 6.748 |
| Personal Pronouns | 9.94(1.12) | 10.78(0.90) | 0.84 | 5.859 | <.001 | 1.125 | 0.558 |
| Negation | 0.44(0.21) | 0.49(0.24) | 0.05 | 1.426 | 0.155 | 0.110 | -0.018 |
| Auxiliary Verbs | 5.51(0.83) | 5.52(0.82) | 0.01 | 0.151 | 0.880 | 0.248 | -0.212 |
| Standard Verbs | 9.26(1.12) | 9.20(0.92) | -0.06 | -0.397 | 0.692 | 0.228 | -0.342 |
| Adjectives | 7.09(1.00) | 7.01(0.84) | -0.08 | -0.661 | 0.509 | 0.171 | -0.344 |
| Tentative | 1.74(0.36) | 1.64(0.48) | -0.10 | -1.675 | 0.096 | 0.018 | -0.220 |
| Affiliation | 3.47(0.79) | 3.61(0.82) | 0.14 | 1.166 | 0.245 | 0.357 | -0.092 |
| Achievement | 9.33(1.07) | 8.86(0.98) | -0.47 | -3.216 | 0.002 | -0.181 | -0.753 |
| Emotion | 1.10(0.38) | 1.12(0.38) | 0.02 | 0.390 | 0.697 | 0.126 | -0.084 |
| Positive emotion | 1.06(0.36) | 1.07(0.37) | 0.01 | 0.340 | 0.734 | 0.120 | -0.085 |
| Social behavior | 6.84(0.87) | 6.79(0.95) | -0.05 | -0.341 | 0.734 | 0.210 | -0.298 |
| Prosocial behavior | 2.83(0.55) | 2.73(0.59) | -0.10 | -1.318 | 0.189 | 0.053 | -0.266 |
| Politeness | 0.57(0.17) | 0.80(0.19) | 0.23 | 8.795 | <.001 | 0.277 | 0.176 |
| Moralization | 0.64(0.29) | 0.63(0.29) | -0.01 | -0.264 | 0.792 | 0.070 | -0.091 |
| Communication | 1.31(0.37) | 1.25(0.43) | -0.06 | -1.053 | 0.293 | 0.052 | -0.172 |
| Social referents | 10.88(1.20) | 12.23(1.19) | 1.35 | 8.000 | <.001 | 1.687 | 1.020 |
| Reward | 1.45(0.31) | 1.30(0.33) | -0.15 | -3.289 | 0.001 | -0.060 | -0.239 |
| Risk | 0.18(0.11) | 0.18(0.13) | 0.00 | -0.293 | 0.770 | 0.028 | -0.037 |
| Curiosity | 2.36(0.77) | 1.97(0.63) | -0.39 | -3.899 | <.001 | -0.191 | -0.583 |
| Need | 0.47(0.20) | 0.46(0.21) | -0.01 | -0.157 | 0.876 | 0.052 | -0.061 |

*Note.* + = higher score for historically female name. *n_male_* _=_ 100, *n*_female_ = 100.
‡ = no longer significant after Benjamini-Hochberg step-up correction.

*Table S4. Independent samples t tests for prompt C (“kind colleague award”)*

|  | **M(SD)** | |  |  |  | **95% CI** | |
| --- | --- | --- | --- | --- | --- | --- | --- |
| **Variable** | **Male** | **Female** | **F - M** | ***t*** | ***p*** | **UL** | **LL** |
| Word Count | 402.61(29.71) | 403.50(28.88) | 0.89 | 0.215 | 0.830 | 0.310 | -0.249 |
| Include | 0.77(0.38) | 0.72(0.32) | -0.04 | -0.887 | 0.376 | 0.154 | -0.405 |
| Agentic | 1.57(0.54) | 1.36(0.47) | -0.21 | -2.987 | 0.003 | -0.141 | -0.688 |
| Agentic+Include | 1.76(0.60) | 1.55(0.49) | -0.21 | -2.723 | 0.007 | -0.105 | -0.654 |
| Avoid | 1.24(0.43) | 1.60(0.63) | 0.35 | 4.637 | <0.001 | 0.890 | 0.359 |
| Communal | 2.79(0.57) | 3.32(1.04) | 0.53 | 4.454 | <0.001 | 0.869 | 0.335 |
| Communal+Avoid | 3.55(0.64) | 4.18(1.16) | 0.63 | 4.725 | <0.001 | 0.901 | 0.370 |
| Analytic | 74.26(5.64) | 75.36(5.32) | 1.10 | 1.420 | 0.157 | 0.479 | -0.078 |
| Clout | 81.58(6.29) | 84.79(5.38) | 3.21 | 3.878 | <0.001 | 0.800 | 0.261 |
| Personal Pronouns | 9.46(1.07) | 9.77(0.79) | 0.32 | 2.374 | 0.019 | 0.608 | 0.056 |
| Negation | 0.47(0.27) | 0.38(0.25) | -0.10 | -2.681 | 0.008 | -0.099 | -0.648 |
| Auxiliary Verbs | 4.32(0.64) | 4.15(0.86) | -0.17 | -1.578 | 0.116 | 0.056 | -0.500 |
| Standard Verbs | 10.45(1.06) | 10.42(1.01) | -0.03 | -0.189 | 0.851 | 0.253 | -0.306 |
| Adjectives | 5.24(0.96) | 4.70(0.91) | -0.54 | -4.068 | <0.001 | -0.285 | -0.823 |
| Tentative | 1.83(0.49) | 1.76(0.44) | -0.07 | -1.084 | 0.280 | 0.125 | -0.432 |
| Affiliation | 7.12(1.06) | 7.68(1.04) | 0.56 | 3.749 | <0.001 | 0.784 | 0.243 |
| Achievement | 6.47(1.17) | 6.44(0.81) | -0.03 | -0.192 | 0.848 | 0.253 | -0.307 |
| Emotion | 2.61(0.67) | 2.63(0.66) | 0.02 | 0.221 | 0.825 | 0.311 | -0.248 |
| Positive emotion | 1.91(0.64) | 2.00(0.52) | 0.08 | 1.023 | 0.308 | 0.424 | -0.134 |
| Social behavior | 11.30(1.04) | 11.72(1.24) | 0.43 | 2.640 | 0.009 | 0.643 | 0.093 |
| Prosocial behavior | 7.29(0.89) | 7.76(1.12) | 0.48 | 3.343 | <0.001 | 0.733 | 0.189 |
| Politeness‡ | 0.67(0.17) | 0.61(0.21) | -0.06 | -2.139 | 0.034 | -0.023 | -0.576 |
| Moralization | 0.62(0.33) | 0.79(0.34) | 0.17 | 3.528 | <0.001 | 0.756 | 0.214 |
| Communication | 1.23(0.43) | 1.08(0.43) | -0.15 | -2.518 | 0.013 | -0.076 | -0.627 |
| Social referents | 12.09(1.18) | 12.60(1.01) | 0.51 | 3.281 | 0.001 | 0.725 | 0.181 |
| Reward | 1.13(0.34) | 1.20(0.32) | 0.08 | 1.631 | 0.104 | 0.507 | -0.048 |
| Risk | 0.21(0.19) | 0.20(0.17) | -0.01 | -0.300 | 0.764 | 0.237 | -0.322 |
| Curiosity | 1.37(0.44) | 1.33(0.38) | -0.04 | -0.732 | 0.465 | 0.176 | -0.383 |
| Need | 0.47(0.20) | 0.47(0.20) | 0.01 | 0.199 | 0.842 | 0.308 | -0.251 |

*Note.* + = higher score for historically female name. *n_male_* _=_ 100, *n*_female_ = 100.
‡ = no longer significant after Benjamini-Hochberg step-up correction.

*Table S5. Independent samples t tests for prompt A^l^ (“...research position in Colorado…”)*

|  | **M(SD)** | |  |  |  | **95% CI** | |
| --- | --- | --- | --- | --- | --- | --- | --- |
| **Variable** | **Male** | **Female** | **F - M** | ***t*** | ***p*** | **UL** | **LL** |
| Word Count | 449.01(35.06) | 424.61(31.52) | -24.40 | -5.176 | <.001 | -15.102 | -33.697 |
| Include | 1.77(0.46) | 1.69(0.41) | -0.08 | -1.352 | 0.178 | 0.038 | -0.205 |
| Agentic | 2.86(0.52) | 2.99(0.54) | 0.13 | 1.733 | 0.085 | 0.277 | -0.018 |
| Agentic+Include | 3.50(0.63) | 3.55(0.61) | 0.05 | 0.552 | 0.581 | 0.220 | -0.124 |
| Avoid | 0.65(0.22) | 0.65(0.25) | 0.00 | -0.243 | 0.808 | 0.058 | -0.074 |
| Communal | 0.55(0.38) | 0.59(0.39) | 0.04 | 0.839 | 0.402 | 0.153 | -0.062 |
| Communal+Avoid | 1.20(0.46) | 1.23(0.42) | 0.03 | 0.432 | 0.666 | 0.150 | -0.096 |
| Analytic | 78.37(4.15) | 74.51(5.75) | -3.86 | -5.439 | <0.001 | -2.459 | -5.256 |
| Clout | 81.14(4.30) | 86.46(4.41) | 5.32 | 8.639 | <0.001 | 6.538 | 4.108 |
| Personal Pronouns | 9.59(0.78) | 10.65(0.87) | 1.06 | 9.033 | <0.001 | 1.290 | 0.828 |
| Negation | 0.41(0.20) | 0.35(0.17) | -0.06 | -2.580 | 0.011 | -0.016 | -0.118 |
| Auxiliary Verbs | 5.20(0.59) | 5.29(0.84) | 0.09 | 0.904 | 0.367 | 0.295 | -0.109 |
| Standard Verbs | 9.43(0.84) | 9.48(1.04) | 0.05 | 0.436 | 0.664 | 0.322 | -0.205 |
| Adjectives | 7.89(0.88) | 7.46(1.04) | -0.43 | -3.166 | 0.002 | -0.162 | -0.697 |
| Tentative | 1.46(0.39) | 1.64(0.44) | 0.18 | 2.996 | 0.003 | 0.293 | 0.060 |
| Affiliation | 3.28(0.64) | 2.76(0.70) | -0.52 | -5.500 | <0.001 | -0.333 | -0.705 |
| Achievement | 8.25(0.94) | 7.94(0.89) | -0.31 | -2.424 | 0.016 | -0.059 | -0.569 |
| Emotion | 1.21(0.39) | 1.28(0.38) | 0.07 | 1.332 | 0.185 | 0.181 | -0.035 |
| Positive emotion | 1.18(0.38) | 1.25(0.38) | 0.07 | 1.299 | 0.195 | 0.175 | -0.036 |
| Social behavior | 6.75(0.75) | 6.27(0.86) | -0.48 | -4.187 | <0.001 | -0.254 | -0.705 |
| Prosocial behavior | 3.04(0.47) | 2.51(0.57) | -0.53 | -7.166 | <0.001 | -0.385 | -0.678 |
| Politeness | 0.54(0.12) | 0.58(0.28) | 0.04 | 1.134 | 0.259 | 0.095 | -0.026 |
| Moralization | 0.47(0.22) | 0.58(0.29) | 0.11 | 3.013 | 0.003 | 0.182 | 0.038 |
| Communication | 1.65(0.40) | 1.60(0.46) | -0.05 | -0.956 | 0.340 | 0.062 | -0.178 |
| Social referents | 9.85(0.82) | 11.24(1.02) | 1.39 | 10.664 | <0.001 | 1.657 | 1.140 |
| Reward | 0.70(0.32) | 0.62(0.34) | -0.08 | -1.745 | 0.083 | 0.011 | -0.174 |
| Risk | 0.18(0.10) | 0.19(0.13) | 0.01 | 1.164 | 0.246 | 0.050 | -0.013 |
| Curiosity | 4.41(0.72) | 3.45(0.58) | -0.96 | -10.320 | <0.001 | -0.773 | -1.138 |
| Need | 0.53(0.17) | 0.57(0.19) | 0.04 | 1.821 | 0.070 | 0.097 | -0.004 |

*Note.* + = higher score for historically female name. *n_male_* _=_ 100, *n*_female_ = 100.
‡ = no longer significant after Benjamini-Hochberg step-up correction.

*Table S6. Independent samples t tests for prompt B^l^ (“...outstanding biological scientist…”)*

|  | **M(SD)** | |  |  |  | **95% CI** | |
| --- | --- | --- | --- | --- | --- | --- | --- |
| **Variable** | **Male** | **Female** | **F - M** | ***t*** | ***p*** | **UL** | **LL** |
| Word Count | 447.77(37.83) | 480.77(60.25) | 33.00 | 4.638 | <0.001 | 47.030 | 18.970 |
| Include | 1.96(0.48) | 2.16(0.56) | 0.21 | 2.827 | 0.005 | 0.353 | 0.063 |
| Agentic | 2.87(0.56) | 3.53(0.68) | 0.66 | 7.503 | <.001 | 0.833 | 0.486 |
| Agentic+Include | 3.44(0.71) | 4.21(0.89) | 0.77 | 6.770 | <.001 | 0.994 | 0.545 |
| Avoid | 0.53(0.21) | 0.51(0.16) | -0.02 | -0.714 | 0.476 | 0.034 | -0.072 |
| Communal | 0.23(0.22) | 0.40(0.28) | 0.17 | 4.573 | <0.001 | 0.233 | 0.092 |
| Communal+Avoid | 0.77(0.32) | 0.91(0.30) | 0.14 | 3.227 | 0.001 | 0.229 | 0.055 |
| Analytic | 81.04(5.00) | 78.78(4.40) | -2.26 | -3.382 | 0.001 | -0.940 | -3.569 |
| Clout | 63.68(6.44) | 68.45(6.42) | 4.77 | 5.237 | <0.001 | 6.558 | 2.970 |
| Personal Pronouns | 7.11(0.72) | 7.73(0.92) | 0.62 | 5.301 | <0.001 | 0.853 | 0.390 |
| Negation | 0.63(0.23) | 0.65(0.27) | 0.02 | 0.819 | 0.414 | 0.098 | -0.041 |
| Auxiliary Verbs | 5.48(0.68) | 5.32(1.04) | -0.16 | -1.272 | 0.205 | 0.087 | -0.403 |
| Standard Verbs | 8.75(0.91) | 8.31(1.29) | -0.44 | -2.804 | 0.006 | -0.131 | -0.753 |
| Adjectives | 8.54(0.87) | 8.15(0.81) | -0.39 | -3.289 | 0.001 | -0.156 | -0.625 |
| Tentative | 1.43(0.54) | 1.21(0.44) | -0.22 | -3.162 | 0.002 | -0.082 | -0.356 |
| Affiliation | 3.44(0.71) | 3.50(0.65) | 0.06 | 0.612 | 0.541 | 0.249 | -0.131 |
| Achievement | 7.87(0.81) | 8.54(0.80) | 0.67 | 5.828 | <.001 | 0.887 | 0.438 |
| Emotion | 0.96(0.33) | 0.96(0.37) | 0.00 | 0.032 | 0.974 | 0.099 | -0.096 |
| Positive emotion | 0.93(0.31) | 0.93(0.34) | 0.00 | -0.152 | 0.879 | 0.085 | -0.099 |
| Social behavior | 6.28(0.81) | 6.64(1.02) | 0.36 | 2.730 | 0.007 | 0.613 | 0.099 |
| Prosocial behavior | 3.00(0.57) | 2.99(0.67) | -0.01 | -0.052 | 0.959 | 0.170 | -0.179 |
| Politeness | 0.53(0.12) | 0.47(0.14) | -0.06 | -3.075 | 0.002 | -0.020 | -0.093 |
| Moralization | 0.48(0.25) | 0.53(0.24) | 0.05 | 1.486 | 0.139 | 0.119 | -0.017 |
| Communication | 0.92(0.33) | 1.11(0.31) | 0.19 | 4.061 | <0.001 | 0.272 | 0.094 |
| Social referents | 7.87(0.81) | 8.93(0.87) | 1.06 | 8.878 | <0.001 | 1.295 | 0.824 |
| Reward | 1.20(0.32) | 1.23(0.28) | 0.03 | 0.675 | 0.500 | 0.111 | -0.055 |
| Risk | 0.19(0.12) | 0.17(0.11) | -0.02 | -0.936 | 0.350 | 0.017 | -0.048 |
| Curiosity | 6.35(0.72) | 5.40(0.57) | -0.95 | -10.355 | <0.001 | -0.767 | -1.128 |
| Need | 0.37(0.17) | 0.34(0.13) | -0.03 | -1.785 | 0.076 | 0.004 | -0.081 |

*Note.* + = higher score for historically female name. *n_male_* _=_ 100, *n*_female_ = 100.
‡ = no longer significant after Benjamini-Hochberg step-up correction.

*Table S7. Independent samples t tests for prompt C^l^ (“...hardworking and compassionate colleague…”)*

|  | **M(SD)** | |  |  |  | **95% CI** | |
| --- | --- | --- | --- | --- | --- | --- | --- |
| **Variable** | **Male** | **Female** | **F - M** | ***t*** | ***p*** | **UL** | **LL** |
| Word Count | 408.22(32.05) | 428.82(53.68) | 20.60 | 3.295 | 0.001 | 32.946 | 8.254 |
| Include | 1.16(0.36) | 1.46(0.42) | 0.30 | 5.424 | <0.001 | 0.409 | 0.191 |
| Agentic | 2.17(0.35) | 2.39(0.39) | 0.22 | 4.227 | <0.001 | 0.326 | 0.118 |
| Agentic+Include | 2.49(0.40) | 3.00(0.51) | 0.51 | 7.840 | <0.001 | 0.637 | 0.381 |
| Avoid | 3.65(0.45) | 3.78(0.51) | 0.13 | 2.030 | 0.004 | 0.272 | 0.004 |
| Communal | 2.25(0.33) | 2.38(0.36) | 0.13 | 2.607 | 0.010 | 0.224 | 0.031 |
| Communal+Avoid | 4.20(0.49) | 4.37(0.49) | 0.17 | 2.344 | 0.020 | 0.299 | 0.026 |
| Analytic | 70.75(5.42) | 75.55(5.75) | 4.80 | 6.070 | <0.001 | 6.351 | 3.236 |
| Clout | 83.03(6.09) | 82.22(6.69) | -0.81 | -0.901 | 0.369 | 0.969 | -2.598 |
| Personal Pronouns | 9.62(0.69) | 8.43(0.98) | -1.19 | -9.920 | <0.001 | -0.955 | -1.429 |
| Negation | 0.56(0.14) | 0.55(0.26) | -0.01 | -0.347 | 0.729 | 0.047 | -0.068 |
| Auxiliary Verbs | 4.32(0.53) | 2.88(0.71) | -1.44 | -16.196 | <0.001 | -1.261 | -1.611 |
| Standard Verbs | 9.68(0.78) | 7.72(1.25) | -1.96 | -13.296 | <0.001 | -1.672 | -2.225 |
| Adjectives | 3.77(0.73) | 4.52(0.72) | 0.75 | 7.372 | <0.001 | 0.952 | 0.550 |
| Tentative | 1.65(0.26) | 1.03(0.28) | -0.62 | -16.184 | <0.001 | -0.536 | -0.685 |
| Affiliation | 8.56(0.73) | 9.00(0.98) | 0.44 | 3.654 | <0.001 | 0.686 | 0.205 |
| Achievement | 8.73(0.84) | 10.25(1.01) | 1.52 | 11.526 | <0.001 | 1.775 | 1.257 |
| Emotion | 1.64(0.50) | 1.89(0.55) | 0.25 | 3.335 | 0.001 | 0.395 | 0.101 |
| Positive emotion | 1.12(0.50) | 1.47(0.46) | 0.35 | 5.098 | <0.001 | 0.480 | 0.212 |
| Social behavior | 10.70(0.78) | 11.61(1.11) | 0.91 | 6.689 | <0.001 | 1.176 | 0.640 |
| Prosocial behavior | 7.11(0.77) | 7.14(0.78) | 0.03 | 0.253 | 0.801 | 0.243 | -0.188 |
| Politeness | 0.51(0.07) | 0.58(0.17) | 0.07 | 3.875 | <0.001 | 0.107 | 0.035 |
| Moralization | 1.45(0.31) | 1.92(0.48) | 0.47 | 8.179 | <0.001 | 0.578 | 0.353 |
| Communication | 0.63(0.22) | 1.01(0.40) | 0.38 | 8.364 | <0.001 | 0.468 | 0.289 |
| Social referents | 12.26(0.92) | 11.62(1.28) | -0.64 | -4.086 | <0.001 | -0.333 | -0.955 |
| Reward | 1.27(0.30) | 1.68(0.26) | 0.41 | 10.147 | <0.001 | 0.481 | 0.324 |
| Risk | 0.23(0.10) | 0.22(0.10) | -0.01 | -1.133 | 0.258 | 0.012 | -0.045 |
| Curiosity | 0.97(0.41) | 1.30(0.35) | 0.33 | 6.237 | <.001 | 0.444 | 0.231 |
| Need | 0.49(0.22) | 0.35(0.15) | -0.14 | -5.133 | <.001 | -0.086 | -0.193 |

*Note.* + = higher score for historically female name. *n_male_* _=_ 100, *n*_female_ = 100.
‡ = no longer significant after Benjamini-Hochberg step-up correction.
