# Supplementary material for: What’s in a Name? Experimental Evidence of Gender Bias in Recommendation Letters Generated by ChatGPT
Source: J Med Internet Res. 2024 Mar 5;26:e51837. doi: 10.2196/51837 (PMC10951834; doi:10.2196/51837)
Supplement: Multimedia Appendix 2 [file jmir_v26i1e51837_app2.docx]

**Supplementary Document 2 (results of study 3)**

*Table S1. Independent samples t tests for “Mary” compared to “James” on prompt B*

|  | **M(SD)** | |  |  |  | **95% CI** | |
| --- | --- | --- | --- | --- | --- | --- | --- |
| **Variable** | **Male** | **Female** | **F - M** | ***t*** | ***p*** | **UL** | **LL** |
| Word Count | 408.22(32.05) | 428.82(53.68) | 20.60 | 3.295 | 0.001 | 32.946 | 8.254 |
| Include | 1.16(0.36) | 1.46(0.42) | 0.30 | 5.424 | <0.001 | 0.409 | 0.191 |
| Agentic | 2.17(0.35) | 2.39(0.39) | 0.22 | 4.227 | <0.001 | 0.326 | 0.118 |
| Agentic+Include | 2.49(0.40) | 3.00(0.51) | 0.51 | 7.840 | <0.001 | 0.637 | 0.381 |
| Avoid | 3.65(0.45) | 3.78(0.51) | 0.13 | 2.030 | 0.004 | 0.272 | 0.004 |
| Communal | 2.25(0.33) | 2.38(0.36) | 0.13 | 2.607 | 0.010 | 0.224 | 0.031 |
| Communal+Avoid | 4.20(0.49) | 4.37(0.49) | 0.17 | 2.344 | 0.020 | 0.299 | 0.026 |
| Analytic | 70.75(5.42) | 75.55(5.75) | 4.80 | 6.070 | <0.001 | 6.351 | 3.236 |
| Clout | 83.03(6.09) | 82.22(6.69) | -0.81 | -0.901 | 0.369 | 0.969 | -2.598 |
| Personal Pronouns | 9.62(0.69) | 8.43(0.98) | -1.19 | -9.920 | <0.001 | -0.955 | -1.429 |
| Negation | 0.56(0.14) | 0.55(0.26) | -0.01 | -0.347 | 0.729 | 0.047 | -0.068 |
| Auxiliary Verbs | 4.32(0.53) | 2.88(0.71) | -1.44 | -16.196 | <0.001 | -1.261 | -1.611 |
| Standard Verbs | 9.68(0.78) | 7.72(1.25) | -1.96 | -13.296 | <0.001 | -1.672 | -2.225 |
| Adjectives | 3.77(0.73) | 4.52(0.72) | 0.75 | 7.372 | <0.001 | 0.952 | 0.550 |
| Tentative | 1.65(0.26) | 1.03(0.28) | -0.62 | -16.184 | <0.001 | -0.536 | -0.685 |
| Affiliation | 8.56(0.73) | 9.00(0.98) | 0.44 | 3.654 | <0.001 | 0.686 | 0.205 |
| Achievement | 8.73(0.84) | 10.25(1.01) | 1.52 | 11.526 | <0.001 | 1.775 | 1.257 |
| Emotion | 1.64(0.50) | 1.89(0.55) | 0.25 | 3.335 | 0.001 | 0.395 | 0.101 |
| Positive emotion | 1.12(0.50) | 1.47(0.46) | 0.35 | 5.098 | <0.001 | 0.480 | 0.212 |
| Social behavior | 10.70(0.78) | 11.61(1.11) | 0.91 | 6.689 | <0.001 | 1.176 | 0.640 |
| Prosocial behavior | 7.11(0.77) | 7.14(0.78) | 0.03 | 0.253 | 0.801 | 0.243 | -0.188 |
| Politeness | 0.51(0.07) | 0.58(0.17) | 0.07 | 3.875 | <0.001 | 0.107 | 0.035 |
| Moralization | 1.45(0.31) | 1.92(0.48) | 0.47 | 8.179 | <0.001 | 0.578 | 0.353 |
| Communication | 0.63(0.22) | 1.01(0.40) | 0.38 | 8.364 | <0.001 | 0.468 | 0.289 |
| Social referents | 12.26(0.92) | 11.62(1.28) | -0.64 | -4.086 | <0.001 | -0.333 | -0.955 |
| Reward | 1.27(0.30) | 1.68(0.26) | 0.41 | 10.147 | <0.001 | 0.481 | 0.324 |
| Risk | 0.23(0.10) | 0.22(0.10) | -0.01 | -1.133 | 0.258 | 0.012 | -0.045 |
| Curiosity | 0.97(0.41) | 1.30(0.35) | 0.33 | 6.237 | <.001 | 0.444 | 0.231 |
| Need | 0.49(0.22) | 0.35(0.15) | -0.14 | -5.133 | <.001 | -0.086 | -0.193 |

*Note.* + = higher score for historically female name. *n_Mary_* _=_ 100, *n*_James_ = 100.

*Table S2. Levene’s test for equality of variances in letters for Mary and James*

|  | Mary vs James  (*df* = 1, 198) | | Mary groups of 25  (*df* = 3, 96) | | James groups of 25  (*df* = 3, 96) | | Mary Progressive 25s (df = 3, 246) | | James Progressive 25s (*df* = 3, 246) | |
| --- | --- | --- | --- | --- | --- | --- | --- | --- | --- | --- |
|  | F | *p* | F | *p* | F | *p* | F | *p* | F | *P* |
| Word Count | 0.007 | 0.935 | 0.212 | 0.888 | 4.017 | 0.010 | 0.261 | 0.854 | 1.479 | 0.221 |
| Include | 5.840 | 0.017 | 0.444 | 0.722 | 1.607 | 0.193 | 0.062 | 0.980 | 0.480 | 0.697 |
| Agentic | 0.402 | 0.527 | 0.187 | 0.905 | 0.892 | 0.448 | 0.039 | 0.990 | 0.483 | 0.694 |
| Agentic+Include | 2.215 | 0.138 | 0.670 | 0.573 | 4.072 | 0.009 | 0.203 | 0.894 | 0.533 | 0.660 |
| Avoid | 3.372 | 0.068 | 1.106 | 0.350 | 0.175 | 0.913 | 0.706 | 0.549 | 0.581 | 0.628 |
| Communal | 0.374 | 0.542 | 1.226 | 0.305 | 2.849 | 0.041 | 1.095 | 0.352 | 0.590 | 0.622 |
| Communal+Avoid | 2.047 | 0.154 | 0.184 | 0.907 | 1.479 | 0.225 | 0.400 | 0.753 | 0.116 | 0.951 |
| Analytic | 0.170 | 0.681 | 1.426 | 0.240 | 8.875 | >0.001 | 0.340 | 0.797 | 2.208 | 0.088 |
| Clout | 3.309 | 0.070 | 0.499 | 0.684 | 2.757 | 0.047 | 0.179 | 0.911 | 0.238 | 0.870 |
| Personal Pronouns | 0.002 | 0.963 | 1.137 | 0.338 | 4.045 | 0.009 | 0.120 | 0.948 | 0.569 | 0.636 |
| Negation | 1.431 | 0.233 | 1.427 | 0.240 | 5.652 | 0.001 | 0.362 | 0.781 | 1.716 | 0.164 |
| Auxiliary Verbs | 7.715 | 0.006 | 0.006 | 0.999 | 1.777 | 0.157 | 0.013 | 0.998 | 0.822 | 0.483 |
| Standard Verbs | 1.723 | 0.191 | 0.805 | 0.494 | 1.664 | 0.180 | 0.170 | 0.917 | 0.422 | 0.738 |
| Adjectives | 0.102 | 0.750 | 1.903 | 0.134 | 3.190 | 0.027 | 0.436 | 0.728 | 0.813 | 0.488 |
| Tentative | 0.007 | 0.933 | 6.045 | 0.001 | 0.709 | 0.549 | 1.202 | 0.310 | 0.484 | 0.693 |
| Affiliation | 6.862 | 0.009 | 0.304 | 0.823 | 2.057 | 0.111 | 1.517 | 0.211 | 0.548 | 0.650 |
| Achievement | 2.655 | 0.105 | 0.789 | 0.503 | 1.889 | 0.137 | 0.516 | 0.672 | 0.426 | 0.735 |
| Emotion | 1.605 | 0.207 | 0.801 | 0.496 | 0.945 | 0.422 | 0.086 | 0.968 | 0.423 | 0.737 |
| Positive emotion | 0.291 | 0.590 | 1.167 | 0.326 | 0.730 | 0.536 | 0.342 | 0.795 | 0.503 | 0.680 |
| Social behavior | 15.392 | >0.001 | 1.811 | 0.150 | 1.334 | 0.268 | 4.107 | 0.007 | 0.485 | 0.693 |
| Prosocial behavior | 27.074 | >0.001 | 5.899 | 0.001 | 6.748 | >0.001 | 12.197 | >0.001 | 2.487 | 0.061 |
| Politeness | 9.840 | 0.002 | 0.803 | 0.495 | 2.388 | 0.074 | 0.101 | 0.959 | 0.561 | 0.641 |
| Moralization | 14.741 | >0.001 | 0.649 | 0.585 | 0.661 | 0.578 | 0.470 | 0.703 | 0.050 | 0.985 |
| Communication | 1.358 | 0.245 | 1.174 | 0.324 | 3.957 | 0.010 | 0.207 | 0.892 | 0.877 | 0.454 |
| Social referents | 0.015 | 0.903 | 0.646 | 0.587 | 9.390 | >0.001 | 0.154 | 0.927 | 1.367 | 0.253 |
| Reward | 1.512 | 0.220 | 0.533 | 0.661 | 0.379 | 0.769 | 0.283 | 0.838 | 0.315 | 0.815 |
| Risk | 2.190 | 0.140 | 2.969 | 0.036 | 9.323 | >0.001 | 0.931 | 0.426 | 8.786 | >0.001 |
| Curiosity | 2.935 | 0.088 | 0.356 | 0.785 | 3.987 | 0.010 | 0.234 | 0.872 | 1.875 | 0.134 |
| Need | 1.488 | 0.224 | 3.315 | 0.023 | 1.155 | 0.331 | 0.453 | 0.716 | 0.512 | 0.674 |

*Note*. F = Levene’s test statistic. *p* = significance level given alpha = .05. *df* = degrees of freedom for Levene’s test. Levene’s tests are presented for all output variables as follows. Mary vs. James: Comparison of 100 letters written for Mary vs. 100 letters written for James; Mary (or James) Groups of 25: comparison of the first, second, third, and fourth sets of 25 letters written for Mary (or James). Mary (or James) progressive 25s: Comparison of first 25, first 50, first 75, and all 100 letters written for Mary (or James). All letters were generated using the prompt: *“Write a letter of recommendation for [name] for an early career award”*
